# Factors Associated with Diabetic Retinopathy in Chinese Patients with Type 2 Diabetes Mellitus

**DOI:** 10.1155/2012/157940

**Published:** 2012-07-10

**Authors:** Bin-Bin He, Li Wei, Yun-Juan Gu, Jun-Feng Han, Ming Li, Yu-Xiang Liu, Yu-Qian Bao, Wei-Ping Jia

**Affiliations:** Shanghai Municipal Key Laboratory on Diabetes, Department of Endocrinology and Metabolism, Shanghai Diabetes Institute, and Shanghai Municipal Clinical Center on Diabetes, Shanghai Jiaotong University Affiliated Sixth People's Hospital, Shanghai 200233, China

## Abstract

*Objective.* To investigate the risk factors of DR in Chinese T2DM patients. *Methods.* 2009 patients with T2DM were included in this cross-sectional study. All patients underwent eye examination, and the DR stage was defined by an ophthalmologist. Correlation analysis was performed to evaluate the relation between DR and clinical variables. Logistic regression models were used to assess risk for those factors associated with DR. *Results.* A total of 597 T2DM patients (29.7%) had DR, of which 548 (27.3%) were nonproliferative diabetic retinopathy and 49 (2.4%) were proliferative diabetic retinopathy. Positive correlations were found between DR and duration of diabetes, systolic blood pressure (SBP), diastolic blood pressure, glycated hemoglobin, glycated albumin, 24 hurinary albumin excretion, peripheral atherosclerosis (PA), diabetes nephropathy (DN), diabetic peripheral neuropathy, and anemia. Negative correlations were found between DR and C-peptide and glomerular filtration rate. Logistic regression analysis revealed that duration of diabetes, SBP, DN, anemia, PA, and C-peptide were each independent risk factors of DR. *Conclusion.* The duration of diabetes, SBP, DN, anemia, and PA are positively associated with DR in Chinese T2DM patients, while C-peptide is negatively associated with DR. Monitoring and evaluation of these related factors will likely contribute to the prevention and treatment of DR.

## 1. Introduction

Type 2 diabetes mellitus (T2DM) is one of the fastest growing diseases in China and has become a major public health challenge. The most common complication of T2DM is diabetic retinopathy (DR), which is a leading cause of preventable blindness in working-aged people. It has been estimated that diabetic individuals are 25 times more likely than their nondiabetic counterparts to suffer severe, permanent vision loss [1]. Although the pathogenesis of DR is still not fully understood, a number of large, multicenter studies in Western populations have identified risk factors of disease onset and progression [2–6]. The Australian Diabetes, Obesity, and Lifestyle study (AusDiab) identified duration of diabetes, glycated hemoglobin (HbA1c), and systolic blood pressure (SBP) as risk factors of DR [6]. The US-Based Early Treatment Diabetic Retinopathy Study (ETDRS) demonstrated that reducing elevated blood lipids and treating anemia in T2DM patients could slow the progression of retinopathy [7]. In addition, many cross-sectional and prospective studies have been carried out in Caucasian populations, which have identified several risk factors of DR, including duration of diabetes, SBP, glycemic control, and urinary albumin [4, 6, 8, 9]. Other factors, including body mass index (BMI), serum lipids, and C-peptide, have shown varying results [4, 8, 10]. Since the current estimates of the prevalence and risk factors for DR have mostly been derived from studies of non-Asian populations, the reliability and utility of these factors in Chinese populations remain unconfirmed. Therefore, the present study was designed to determine whether the most recognized DR risk factors in the literature are similar to those in Chinese T2DM patients. These data will not only provide insights into the common and distinctive features of DR in Chinese and Caucasian populations but also identify clinically relevant factors, which may be monitored or therapeutically manipulated to reduce the frequency and severity of DR.

## 2. Materials and Methods

### 2.1. Study Participants

A retrospective, cross-sectional analysis was performed in T2DM patients who had been admitted to the Endocrinology Department of Shanghai Jiaotong University Affiliated Sixth People's Hospital from January 2008 to December 2009. All of the patients had been admitted specifically for management of their diabetes. Diagnosis of diabetes was performed according to the 1999 World Health Organization criteria. Patients were excluded according to the presence of type 1 diabetes mellitus, any other non-type 2 diabetes (such as gestational diabetes), poor general condition, severe infection, acute cerebrovascular disease, recent surgery, impaired cardiac function (III-IV degree according to NYHA classification), or severely impaired hepatic or renal function.

### 2.2. Determination of Variables

Clinical data was extracted from the departments medical records for each patient and included age, gender, duration of diabetes, and previous history of hypertension and dyslipidemia. Patients' medical examinations on the day of admission had included measurements of height, body weight, and blood pressure. BMI was calculated as kg/m^2^. Hypertension was defined as diastolic blood pressure (DBP) ≥ 90 mmHg, SBP ≥ 140 mmHg, or self-reported use of antihypertensive medication. Dyslipidemia was defined as an elevation of total cholesterol (TC) ≥ 6.1 mmol/L and/or of triacylglycerol (TG) ≥ 2.26 mmol/L, or an elevated low-density lipoprotein cholesterol (LDL-C) ≥ 4.14 mmol/L or a decreased high-density lipoprotein cholesterol (HDL-C) < 1.0 mmol/L, according to the American National Cholesterol Education Program (Adult Treatment Panel III) [11]. Coronary heart disease (CHD) was considered to be present if the patient had an ischemic history or ECG abnormality. Cerebral vascular disease (CVD) was diagnosed based on history of ischemic attack or strokes. As a method to diagnose peripheral atherosclerosis (PA), ultrasonography of carotid or lower limb arteries was performed to detect the presence of thickening or plaque on the wall of those blood vessels. Diabetic peripheral neuropathy (DPN) was diagnosed by electromyogram (EMG). Diabetic nephropathy (DN) was indicated by both microalbuminuria (24 h urinary albumin excretion between 30 and 300 mg) and macroalbuminuria (>300 mg in at least two of three measurements). Hyperuricemia/gout was indicated by explicit history or measurements obtained from plasma uric acid (Ua) assay. Glomerular filtration rate (GFR) was measured by radionuclide renal dynamic imaging, after intravenous injection of ^99^mTc-diethylene-triamine-pentaacetic acid (^99^mTc-DTPA). The ^99^mTc-DTPA renal dynamic imaging (modified Gate's method) was measured by a Siemens Signature e.cam SPECT (General Electric Medical Systems, Waukesha, WI, USA). Anemia was defined according to Chinese anemia sex-based criteria, which is hemoglobin (Hb) concentration of <12 g/dL for men and <11 g/dL for women. In addition, the patients with anemia were further subdivided into two groups: mild anemia (Hb ≥ 9 g/dL) and moderate and severe anemia (Hb < 9 g/dL).

### 2.3. Laboratory Assays

Fasting blood samples were drawn before breakfast on the second day of hospitalization for measurements of fasting plasma glucose (FPG), HbA1c, glycated albumin (GA), C-peptide, Ua, TC, TG, LDL-C, and HDL-C. The 2 h postprandial glucose (2 h PG) levels were drawn after a mixed meal. The plasma glucose concentration was measured using a glucose oxidase method (Automatic Biochemistry Analyzer; Beckman Coulter, USA). Serum lipids and renal function indices were determined by enzymatic procedures using an autoanalyzer (7600-020; Hitachi, Tokyo, Japan). Serum C-peptide was determined by radioimmunoassay (Linco, USA). HbA1c was estimated by high-performance liquid chromatography using the HLC-723G7 analyzer (Tosoh Corporation, Japan). GA was measured by a liquid enzymatic assay kit (Lucica GA-L; Asahi Kasei Pharma, Japan). The 24 h urinary albumin concentration was determined by nephelometry using an N antiserum to Human Albumin Assay and BN II analyzer (Dade-Behring, USA). Hb was measured using an automated blood analyzer (XE-5000; Sysmex Corp., Japan).

### 2.4. Detection of DR

Fundus photographic imaging was carried out by following a standardized protocol. Both eyes of each participant were photographed with a 45-degree 6.3-megapixel digital nonmydriatic camera (Canon CR6-45NM, Lake Success, NY, USA). According to the International Clinical Diabetic Retinopathy and Diabetic Macular Edema Disease Severity Scales [12], the severity of DR was treated as an ordinal variable: 0: no findings of diabetic retinopathy (NDR); 1: nonproliferative diabetic retinopathy (NPDR, including mild, moderate, and severe NPDR); and 2: proliferative diabetic retinopathy (PDR). Patients whose fundus diseases were of unequal severity in both eyes were staged according to the more severe one. NPDR and PDR were, respectively, considered as the early and late stage of DR; thus, in this analysis the two categories were combined as a single group (DR group) for both 2-group comparison and logistic regression.

### 2.5. Statistical Analysis

All statistical analyses were carried out using the Statistical Package for the Social Sciences software version 16.0 (SPSS Inc., Chicago, IL). Numerical variables of normal distribution were expressed as mean ± SD, while those of nonnormal distribution were expressed as median (quartile). One-way ANOVA and *t-*test were performed to compare means of normally distributed variables. The Mann-Whitney *U* test was used to evaluate nonnormally distributed variables. Spearman's rank correlation analysis was also used to examine the association of clinical variables with DR. Chi-square (*χ* ^2^) test was used to analyze categorical variables. Trend *χ* ^2^ test was used to compare proportions among groups. The significance level for the above statistical analyses was set as **α** = 0.05, and a *P* value < 0.05 (two-tailed) was considered as statistically significant. 

In this study, binary logistic regression was primarily used to estimate the association of independent factors and DR by Wald and *P* value, for which <0.05 was considered as statistically significant. The ability of each independent factor to relate to DR was determined by partial regression coefficient (B) and odds ratio (OR = EXP(B)), along with its 95% confidence interval (95% CI). OR above 1 represented positive correlation, and OR below 1 represented negative correlation.

## 3. Results

### 3.1. Subjects' Clinical Characteristics and Comorbidities

According to the inclusion and exclusion criteria mentioned above, a total of 2009 T2DM patients were included in this analysis. There were 1145 (57%) males and 864 (43%) females, with mean age of 59.69 ± 12.28 years and diabetic duration of 8.05 ± 6.71 years. Of all the subjects, 597 (29.7%) were diagnosed with DR, of which 548 (27.3%) had NPDR and 49 (2.4%) had PDR. Comparisons between the two groups (NDR versus DR) revealed a significantly higher prevalence of anemia, hypertension, DN, DPN, and PA in the DR group. In contrast, no differences were found between the two groups for dyslipidemia, CHD, CVD, and hyperuricemia/gout. PA, DN, DPN, hypertension, and anemia were positively correlated with DR (Table 1).

### 3.2. Comparisons of Clinical Parameters

Comparisons of clinical parameters according to the retinopathy status are shown in Table 2. Patients with DR were found to have significantly longer duration of diabetes and elevated SBP and DBP, but significantly lower levels of Hb and GFR. No differences were observed in serum lipids, including TC, TG, LDL-C, and HDL-C. Patients with DR (NPDR or PDR) tended to have higher HbA1c and GA levels, which reflected the status of blood glucose control, while C-peptide levels were significantly lower than those of the NDR group. The duration of diabetes, SBP, DBP, HbA1c, and GA levels were found to be positively correlated with DR; Hb, HDL, GFR, C-peptide, and GFR were negatively correlated.

### 3.3. Regression Analyses of DR

Age, gender, duration of diabetes, BMI, dyslipidemia, HbA1c, SBP, DBP, C-peptide, anemia, DN, DPN, and PA were entered into a logistic regression model. The results of the logistic regression analyses are displayed in Table 3. There were strong significant associations between the development of DR and duration (OR = 1.083, 95% CI 1.061–1.105), SBP (OR = 1.013, 95% CI 1.006–1.021), presence of DN (OR = 1.614, 95% CI 1.217–2.142), presence of anemia (OR = 1.857, 95% CI 1.225–2.815), and presence of PA (OR = 1.589, 95% CI 1.161–2.176). C-peptide (OR = 0.761, 95% CI 0.669–0.867) was negatively associated with DR.

### 3.4. Relationship between Each Related Factor and the Severity of DR

**Figure 1:**
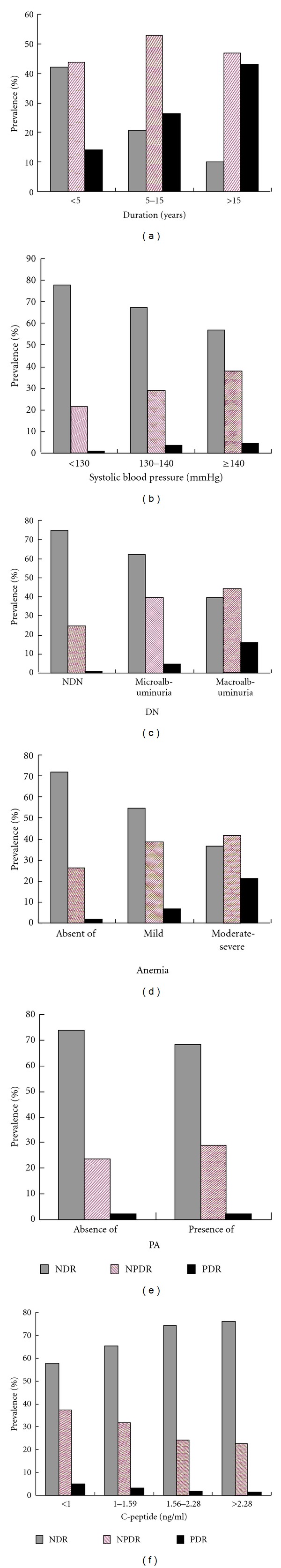
Prevalence of NDR, NPDR, and PDR for the related risk factors. All had *P* value < 0.01 by the trend test.

The relationship between each independent factor associated with DR and the severity of retinopathy was examined. The prevalence of NPDR and PDR was much higher in patients with longer duration of diabetes, higher level of SBP, increased severity of DN, more severe anemia, presence of PA, and lower serum C-peptide quartile (all *P* < 0.01) (Figure 1).

## 4. Discussion

The results of this clinic-based study suggest that independent factors associated with DR in Chinese patients include duration of diabetes, SBP, C-peptide, DN, PA, and anemia. In particular, the severity of DR was found to be increased in conditions of lower C-peptide levels and elevated levels of the other independent factors. 

In our study, the duration of diabetes was found to be significantly higher in patients with DR than in those without, and the longer the duration of diabetes the higher the prevalence of retinopathy. Our findings agree with those from previously published studies in populations of other ethnic groups that also identified duration of diabetes to be an independent risk factor for retinopathy [13–15]. It is presumed that duration of diabetes reflects total glycemic control and risk factor exposure over time. 

HbA1c and GA were found to be significantly higher in DR patients from our Chinese study population. However, they did not retain significance in the final logistic regression analysis. This was different from the results of previous studies [4, 16–19]. This difference may be a result of most of the patients in our study having been admitted to the hospital to treat severely poor glycemic control, and the long-term control of blood glucose might not have been reflected through the levels of HbA1c and GA detected at admission. The large United Kingdom Prospective Diabetes Study (known as the UKPDS) found that every 1% decrease in HbA1c was accompanied by a 24% reduction in the risk of developing DR [20]. Therefore, glycemic control in patients with T2DM was confirmed as very important to lowering a patient's risk for developing DR.

The current study in Chinese patients showed that the prevalence of hypertension was higher in subjects with DR than in those without, and the study found that SBP was positively associated with DR. The UKPDS also found that the incidence of retinopathy was associated with SBP in their largely Caucasian population; specifically, the relative risk for DR was found in the UKPDS to be 1.5 for systolic pressures between 125 and 139 mmHg and 2.8 for systolic pressures higher than 140 mmHg [15]. It is known that DR can be affected by the hemodynamic changes induced by hypertension, such as impaired autoregulation and hyperperfusion [21]. In addition, hypertension independent of hyperglycemia is known to upregulate the expression of vascular endothelial growth factor (VEGF) in retinal endothelial cells and ocular fluids, which can promote DR [22].

Dyslipidemia was not shown in our study population to be associated with retinopathy, which was consistent with the reported results from some of the other studies in the literature [6, 23]. However, this was different from the results of two large prospective studies, the Early Treatment Diabetic Retinopathy study [24] and the Wisconsin Epidemiological study [25]. Both of these found a statistically significant association between elevated serum total cholesterol and LDL-C cholesterol and the severity of retinal hard exudation in patients with DR. Furthermore, subsequent evidence proved the effectiveness of lipid-lowering therapy in ameliorating DR progression [26, 27]. Regression analyses in our study did not find any influence of dyslipidemia on DR, likely due to the poorly controlled lipid levels upon admission of our patients, on which our data was based.

Albuminuria excretion was significantly higher in our patient population with DR than in those without, and GFR was negatively correlated to DR. Further analysis showed that DN was observed to be an independent risk factor for the development of DR, with odds of 1.614 times. Moreover, the severity of DR in patients with T2DM appeared to be aggravated with an increasing severity of DN. Higher risk of concomitant DR might be predicted in patients with DN or renal dysfunction. This could be explained by the fact that both nephropathy and retinopathy are related to endothelial dysfunction-mediated microvascular complications of diabetes mellitus (DM). A number of previous studies have published evidence to support the suggestion that DR and DN progress in a parallel manner; thus, the presence of one is believed to predict the development of the other [10, 28, 29]. Therefore, all patients with DN should be regarded as a high-risk group for their increased risk of developing and the progression of DR, and close monitoring for DR is recommended to prevent irreversible visual loss. 

C-peptide was negatively correlated with DR in our study, indicating it as a protective factor against DR. Our results were consistent with a previous study that also found reduced serum C-peptide to be associated with a higher prevalence of retinopathy in T2DM [23]. Considering the numerous studies that have been conducted on the structure, membrane binding, and biological functions of C-peptide, it seems likely that this protein may indeed play a crucial role in the pathogenesis of DM and its associated complications [30]. Even slightly preserved function of the insulin-producing cells has been shown to have beneficial effects on glucose disposal and metabolic control, thereby decreasing the occurrence of subsequent microvascular complications [31]. C-peptide has also been proven to be an active peptide hormone with potentially important physiological effects [32–35]. For example, C-peptide can stimulate Na^+^-K^+^-ATPase, endothelial nitric oxide synthase activities [35–37], and nuclear factor-*κ*B activation of endothelial cells, all of which are enzyme systems of significant benefit in the prevention and treatment of DR. Furthermore, an experimental study has shown that C-peptide is involved in regulating extracellular matrix (ECM) protein composition, which is one of the characteristic features of DR [38].

In agreement with findings of previous studies [39, 40], we also identified a close relationship of PA and DR in diabetic patients. Atherosclerotic changes in the carotid or lower limb arteries generally reflect systemic atherosclerosis. In addition, vascular endomembrane damage and endothelial dysfunction are considered to be the common initial factors and pathological mechanisms of both macro- and microvascular complications [41].

Anemia was found in our study to be another risk factor of DR in Chinese patients, perhaps because of anemia-induced retinal hypoxia. Individuals with anemia were 1.857 times more likely to develop DR than individuals without anemia. Moreover, the prevalence of PDR was significantly increased in patients with moderate-severe anemia than those with absolutely no detectable anemia. These results are consistent with those reported in the ETDRS, in which low hematocrit was identified as an independent risk factor for the development of high-risk PDR and visual impairment [7]. Another large cross-sectional study showed that the odds ratio of having any retinopathy was twofold higher for individuals with an Hb level of <12 g/dL, as compared with those having an Hb level ≥12 g/dL. In addition, DR patients with low hemoglobin levels had over fivefold increased risk of severe retinopathy, as compared to those with higher hemoglobin levels [42]. These results suggested that subjects with low Hb levels tended to have an increased risk of retinopathy, especially that of the severe form. Other studies have corroborated this finding and have also found evidence of improvement in the DR status following correction of anemia [43–45]. Although much attention has been given to anemia in diabetes in recent years, particularly as it relates to kidney disease [46–50], little attention has been given to low Hb levels as a risk factor for the development or worsening of DR. 

There were several limitations to the present study that should be noted here, as they may affect the generalization of our findings. Because this was a cross-sectional study, the results do not provide definite information on a cause-and-effect relationship. In addition, because our study population was composed solely of patients from a single center, there was unavoidable bias of selection, information, and confounding variables. Prospective and larger studies are required to overcome these limitations. 

## Figures and Tables

**Table 1:** Comparison of clinical characteristics and comorbidities among DR groups: *N* (%).

	Total	NDR	DR	*P*	R*
*N*	2009 (100)	1412 (100)	597 (100)		
Male	1145 (57)	832 (58.9)	313 (52.4)	0.007	−0.060
Female	864 (43)	580 (41.1)	284 (47.6)		
Dyslipidemia	1503 (74.8)	1063 (75.3)	440 (73.7)	0.564	−0.017
Hypertension	1166 (58)	778 (55.1)	388 (65)	0.000	0.092^‡^
Anemia	164 (8.2)	86 (6.1)	78 (13.1)	0.000	0.116^‡^
CHD	122 (6.1)	90 (6.4)	32 (5.4)	0.385	−0.019
CVD	108 (5.4)	78 (5.5)	30 (5.0)	0.650	−0.100
PA	1334 (66.4)	912 (64.6)	422 (70.7)	0.008	0.059^‡^
DN	497 (24.7)	283 (20.0)	214 (35.8)	0.000	0.167^‡^
DPN	866 (43.1)	541 (38.3)	325 (54.4)	0.000	0.149^‡^
Hyperuricemia/ Gout	208 (10.4)	151 (10.7)	57 (9.5)	0.441	−0.017

^∗^ Univariate relation of DR with clinical comorbidities was determined using Spearman's correlation coefficient.

^‡^ *P* < 0.01.

**Table 2:** Comparison of clinical parameters between DR groups.

	Total	NDR	DR	*P*	R*
Age, years	59.69 ± 12.28	59.89 ± 12.81	59.21 ± 10.93	0.227	−0.032
Duration, years	8.05 ± 6.71	6.99 ± 6.29	10.58 ± 6.98	<0.001	0.255^‡^
BMI, kg/m^2^	24.77 ± 3.51	24.84 ± 3.53	24.61 ± 3.45	0.174	−0.028
SBP, mmHg	131.40 ± 17.39	129.53 ± 16.55	135.79 ± 18.51	<0.001	0.164^‡^
DBP, mmHg	79.61 ± 9.68	79.18 ± 9.48	80.62 ± 10.08	0.002	0.068^‡^
Hb, g/L	135 (125~145)	136 (126~146)	132 (122~143)	<0.001	−0.105^‡^
TC, mmol/L	4.75 ± 1.13	4.72 ± 1.10	4.81 ± 1.20	0.149	0.034
TG, mmol/L	1.86 ± 1.65	1.88 ± 1.76	1.79 ± 1.36	0.269	−0.035
HDL-C, mmol/L	1.11 ± 0.32	1.10 ± 0.32	1.13 ± 0.31	0.056	0.052^†^
LDL-C, mmol/L	3.22 ± 1.0	3.21 ± 0.98	3.25 ± 1.03	0.515	0.008
FPG, mmol/L	8.32 ± 2.84	8.29 ± 2.73	8.39 ± 3.09	0.480	−0.007
2 hPG, mmol/L	14.06 ± 4.67	14.05 ± 4.64	14.09 ± 4.76	0.827	0.005
HbA1c, %	8.7 (7.2~10.5)	8.5 (7.1~10.4)	9.1 (7.5~10.7)	0.003	0.068^‡^
GA, %	24 (19~30)	24 (19~30)	25 (20~31)	<0.001	0.09^‡^
C-peptide, ng/mL	1.59 (1.0~2.28)	1.70 (1.11~2.41)	1.35 (0.85~2.04)	<0.001	−0.157^‡^
IMT, mm	0.94 ± 0.71	0.92 ± 0.62	0.99 ± 0.87	0.047	0.049^†^
24 h-urinary albumin, mg	10.92 (5.72~30.62)	9.37 (5.32~23.15)	18.66 (7.25~77.60)	<0.001	0.213^‡^
GFR, mL/min	92.47 ± 25.28	93.60 ± 25.17	89.66 ± 25.37	0.003	−0.069^‡^

Data are mean ± SD or mean (quartile).

^∗^ Univariate relation of DR with clinical parameters were determined using Spearman's correlation coefficient.

^†^ *P* < 0.05, ^‡^ *P* < 0.01*.*

**Table 3:** Logistic-regression-identified factors associated with DR.

Items	*B*	S.E.	Wald	*P*	OR	95% CI of OR	
						Lower boundary	Upper boundary
Duration	0.080	0.011	56.809	0.000	1.083	1.061	1.105
SBP	0.013	0.004	12.848	0.000	1.013	1.006	1.021
DN	0.479	0.144	11.041	0.001	1.614	1.217	2.142
Anemia	0.619	0.218	8.509	0.004	1.857	1.225	2.815
PA	0.463	0.160	8.360	0.004	1.589	1.161	2.176
C-peptide	−0.273	0.066	17.018	0.000	0.761	0.669	0.867
